# Initial Clinical Experience with Ahmed Valve in Romania: Five-Year Patient Follow-Up and Outcomes

**DOI:** 10.3390/bioengineering11080820

**Published:** 2024-08-12

**Authors:** Ramona Ileana Barac, Vasile Harghel, Nicoleta Anton, George Baltă, Ioana Teodora Tofolean, Christiana Dragosloveanu, Laurențiu Flavius Leuștean, Dan George Deleanu, Diana Andreea Barac

**Affiliations:** 1 Department of Ophthalmology, “Carol Davila” University of Medicine and Pharmacy, 050474 București, Romania; ramona.barac@umfcd.ro (R.I.B.); george.balta@drd.umfcd.ro (G.B.); ioana.tofolean@umfcd.ro (I.T.T.); flavius-laurentiu.leustean@drd.umfcd.ro (L.F.L.); dan-george.deleanu@umfcd.ro (D.G.D.); andreeadiana.e.barac@stud.umfcd.ro (D.A.B.); 2 Department of Ophthalmology, University of Medicine and Pharmacy “Grigore T. Popa”, 700115 Iași, Romania; anton.nicoleta1@umfiasi.ro

**Keywords:** glaucoma, Ahmed glaucoma valve implant

## Abstract

Background: Glaucoma is a leading cause of irreversible blindness worldwide and is particularly challenging to treat in its refractory forms. The Ahmed valve offers a potential solution for these difficult cases. This research aims to assess the initial clinical experience with Ahmed valve implantation in Romania, evaluating its effectiveness, associated complications, and overall patient outcomes over a five-year period. Methods: We conducted a prospective study on 50 patients who underwent Ahmed valve implantation due to various types of glaucoma. Patients were monitored at several intervals, up to five years post-surgery. Intraocular pressure and visual acuity were the primary measures of success. Results: On average, patients maintained the intraocular pressure within the targeted range, with the mean intraocular pressure being 17 mmHg 5 years post-surgery. Success, defined as maintaining target intraocular pressure without additional surgery, was achieved in 82% at 1 year, 68% at 3 years, and 60% after 5 years postoperative. Conclusion: Ahmed valve implantation is a viable treatment option for refractory glaucoma, demonstrating significant intraocular pressure reduction and manageable complication rates over a five-year follow-up period. Future research should focus on long-term outcomes and optimization of surgical techniques to further reduce complication rates and improve patient quality of life.

## 1. Introduction

The management of glaucoma, a leading cause of irreversible blindness worldwide, continues to present significant challenges to ophthalmologists. Traditional treatment modalities, including pharmacological therapy and conventional surgical procedures, often fall short of adequately controlling intraocular pressure (IOP) in advanced cases. The advent of innovative surgical interventions, such as the Ahmed glaucoma valve (AGV) implant, offers new hope for patients who struggle with refractory glaucoma (resistant to conventional medical, laser, and surgical treatments aimed at reducing intraocular pressure) [1,2,3,4,5].

This article delves into the initial clinical experiences with the AGV implant, highlighting the outcomes and implications of a recent clinical trial involving a cohort of patients who underwent this procedure. By assessing the effectiveness, safety, and patient outcomes associated with the AGV, this study aims to provide a comprehensive evaluation of its role in the modern therapeutic arsenal against glaucoma. This is the first study conducted in Romania on a group of patients with refractory glaucoma, with a follow-up period of 5 years, providing valuable insights into the long-term effectiveness and safety of the AGV implant in this patient population. Our focus is to thoroughly analyze the socio-demographic characteristics, types of glaucoma, postoperative IOP variations, visual acuity outcomes, and the complications encountered over the extended follow-up period. 

Globally, the AGV has become a pivotal tool in the surgical management of refractory glaucoma due to its effectiveness in reducing IOP and its relatively favorable safety profile. Studies from various regions, including long-term analyses, have consistently demonstrated the AGV’s capability to achieve significant IOP reduction with manageable complication rates [4,5,6]. For instance, Kang et al. (2022) reported favorable long-term outcomes, highlighting both the efficacy and the sustained benefits of AGV implantation [4]. Similarly, Christakis et al. (2017) found comparable results when contrasting the AGV with the Baerveldt implant, reinforcing the AGV’s standing as an effective option for IOP control in refractory cases [7,8].

The study also places its findings in the context of international research, comparing outcomes with significant studies such as the Tube Versus Trabeculectomy (TVT) study and the Primary Tube Versus Trabeculectomy (PTVT) study, both of which provide extensive data on the efficacy and complications associated with tube shunt surgeries [9,10,11,12,13,14]. This comparison is crucial in understanding the broader implications of AGV use and its positioning relative to other surgical options. Furthermore, by documenting the socio-demographic characteristics and types of glaucoma treated, this study aims to contribute to a more nuanced understanding of how these factors influence surgical outcomes and complications.

Our findings are expected to contribute significantly to the global body of knowledge on glaucoma management, offering a unique perspective from a Romanian cohort and adding to the diversity of clinical experiences with the AGV. The insights gained from this study will not only enhance clinical practice in Romania but also provide valuable data for ophthalmologists worldwide who are managing patients with refractory glaucoma.

## 2. Materials and Methods

Data collection for this study was meticulously planned and executed to ensure the accuracy and reliability of the findings. Fifty-eight patients with refractory glaucoma were recruited from the Clinical Hospital for Ophthalmological Emergencies Bucharest between 2014 and 2018. All patients provided written informed consent prior to participation. Patients had different forms of uncontrolled glaucoma, either by medication or surgery.

The choice of AGV over other treatment options was based on several considerations. It has consistently demonstrated significant reductions in IOP across various studies and patient populations [6,7]. Its non-valved mechanism allows for a more controlled release of aqueous humor, reducing the risk of postoperative hypotony [8]. Long-term studies have demonstrated the sustained benefits of AGV implantation in maintaining reduced IOP levels and minimizing complications over extended periods. Also, it has proven to be effective in managing refractory glaucoma, including cases with neovascular glaucoma and other challenging conditions. This makes it a preferred option for patients who have not responded to conventional treatments [9,10,11,12,13,14].

During the study, three patients dropped out due to death, and five patients missed follow-up visits. For patients who missed visits, the last observation carried forward method was employed to handle missing data, ensuring the robustness of the analysis. The criteria for inclusion are as follows:

- Indication for AGV implant;
- Age ≥ 18;
- Consent of the patient to participate in the study;
- Refractory glaucoma;
- Compliance with follow-ups for a 5-year period.

The criteria for success are as follows:

- IOP less than 22 mmHg and greater than 5 mmHg (with or without medication);
- Decrease in visual acuity by no more than two lines at Snellen optotype;
- Solved postoperative complications (if any occurred);
- had a functional valve on follow-up.

The sample size for this study was calculated based on several critical parameters to ensure sufficient power to detect meaningful clinical differences and to achieve statistically significant results. The following parameters were used for determining the sample size:

- The anticipated difference in the primary outcome measure between baseline and post-intervention.
- The effect size was estimated based on previous studies and clinical expectations of the AGV effectiveness.
- An estimate of the proportion of participants who might drop out of the study or miss follow-up visits.
- Based on similar studies, an anticipated dropout rate of approximately 10% was factored into the sample size calculation to account for potential loss to follow-up.
- The prevalence of refractory glaucoma and the availability of eligible patients within the recruitment period.
- Feasibility and resource constraints were also considered to ensure the study could be practically conducted within the specified timeframe.

Before surgery, a blood sample was taken for laboratory tests. The tests included standard assessments of patients’ general health. The approximate amount of blood taken did not exceed 41 mL (3 tablespoons), which corresponds to the total volume of blood taken throughout the study. All information obtained from the samples was kept confidential.

**Figure 1:**
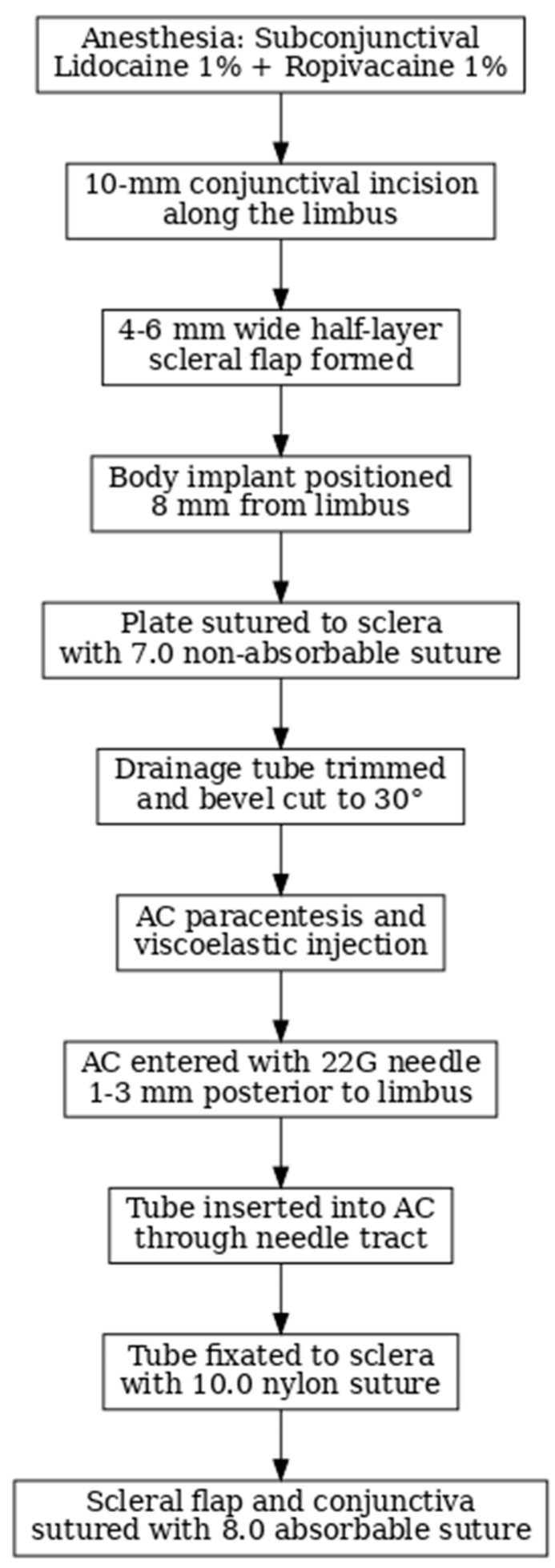
Ahmed glaucoma valve implant: surgery steps.

All AGV implantation procedures were performed by the same surgeon and using identical processes, including subconjunctival anesthesia, during the study period. All patients were anesthetized with subconjunctival anesthesia, using Lidocaine 1% mixed with Ropivacaine 1%. A 10 mm conjunctival incision was created along the limbus to construct a conjunctival flap, and a 4–6 mm wide half-layer scleral flap was formed. The body implant was positioned 8 mm from the limbus, outside the limbal healing space. The plate was then sutured to the sclera with a 7.0 non-absorbable suture. The drainage tube was trimmed to permit a 2–3 mm insertion in the AC and was bevel cut to an angle of 30° to facilitate AC entering. An AC paracentesis was performed, and a viscoelastic substance was injected to increase the space. The AC was then entered 1–3 mm posteriorly to the corneoscleral limbus with a 22G needle. The tube was inserted into the AC through the needle tract. An additional 10.0 nylon suture was placed to fixate the tube to the sclera. Afterward, both the scleral flap and conjunctiva were then sutured with 8.0 absorbable suture. We did not use Mitomycin as it is not approved by the National Agency for Medicines and Medical Devices in Romania. We did not use any other antimetabolite at implantation to avoid increasing the risk of denudation and valve expulsion. 5-fluorouracil was used per secundum in the patients who required needling [10,13]. All patients received similar postoperative topical medications: 0.5% chloramphenicol and 0.2% betamethasone four times daily for one month. The model selected for implantation was the FP7 in all patients in the superotemporal quadrant (Figure 1).

The information collected was introduced into a computerized database. Excel was used for data entry and processing, and statistical analyses were conducted using SAS Enterprise Guide 9.4M7 software. We used various statistical analyses, statistical tests, t-student test for hypothesis testing, descriptive analysis, averaging, calculation of absolute and relative frequency in percentages, correlations (using Pearson test), and correlation ratio to interpret the intensity of independent variables as compared to dependent variable for linear regression models, each variable validation, graphics, and histogram frequencies. The results are reported based on the total number of cases remaining in the study.

We measured the visual acuity with the best correction using the Snellen optotype. Intraocular pressure was measured using the Goldmann tonometer with topical anesthesia. We noted the antiglaucoma treatment that patients use with each measurement. Visual field examination results were not tracked as a parameter because the target was to decrease IOP. Moreover, the patients included in the study had mostly significant low acuity and visual field loss at the beginning of the study; thus, substantial visual field changes were not anticipated as primary outcomes. The primary intervention goal was to stabilize and lower IOP to prevent further progression of glaucoma.

We have received ethical approval from the Clinical Emergency Eye Hospital Bucharest Ethic Committee for this study.

## 3. Results

- Analysis of socio-demographic characteristics

The group comprised 50 patients with an average age of 51.7 years old, with the youngest being 18 and the oldest being 84 years old. We noticed that 40% of the patients had elementary studies, and 60% had at least a professional qualification. There is a discrepancy between the level of professional education (62% of the patients have a qualification) and the job of the adult patients. Only six (12%) patients were employed, and none were employers or workers on their own.

- Distribution of patients by glaucoma type

Most of the patients had an admission diagnostic of glaucoma secondary to vitreoretinal surgery (24%). The cause of this phenomenon is the fact that the clinic where the surgeries were performed has a high number of interventions on the posterior pole (Table 1).

In second place as ethology in our study, we have primary glaucoma with an open angle. In 10 patients (20%) with primary open-angle glaucoma that was not amendable with medical treatment or other surgical interventions and had a progression to atrophy, we implanted AGV.

The third place stands for neovascular glaucoma, with eight patients (16%). Neovascular glaucoma is only in third place because of the use of anti-VEGF agents in the last few years. This has led to a decrease in the number of patients with neovascular glaucoma to whom an AGV was implanted [15,16].

- Intraocular pressure variation after surgery

The targeted intraocular pressure was below 22 mmHg and above 5 mmHg. Hypotonia is defined as a decrease in the intraocular pressure below 5 mmHg. In the group of patients that we examined, the pressure was measured before surgery, one day, one month, 3 months and 6 months, one year, three years, and five years after surgery and in all patients included in the study when they presented in the hospital for different problems. We calculated the mean values of the intraocular pressure at every step of the follow-up. One day after surgery, only one patient had hypotonia and a small anterior chamber. A visco-elastic substance was injected into the anterior chamber, and the pressure became normal in the following days. During the study, there were no other cases of hypotonia.

The very small number of patients with hypotonia after the implantation of AGV is explained by the fact that the technique used implies covering the tube with a scleral flap. This decreased risk was also described by other authors when using the scleral flap [17,18]. Because of the elastomeric membrane that functions as a valve, the system remains closed when the intraocular pressure decreases below 8 mmHg. There can be losses next to the tube or if the tube is punctured accidentally in the extra cameral portion; in these instances, the valve works, but hypotonia ensues because of the losses outside the valve [9,10,11,12,13,14,15,16,17,18,19,20,21,22].

One patient had an intraocular pressure of 30 mmHg one day after surgery, and this happened because of a blockage of the tube by vitreous. The pressure did not decrease after administration of specific drugs locally or systemically, so we decided to practice anterior vitrectomy.

The IOP a day after surgery had a mean value of 12 mmHg. In patients with an intraocular pressure above 22 mmHg, antiglaucoma medication was administered, or massage of the eye was made, or needling or surgical excision of the fibrous capsule was intended.

In most patients with hypertonia, this was caused by the formation of a capsule of fibrous tissue around the body of the valve with modified collagen because of the presence of the aqueous humor under the tenon, between the tenon and the sclera. This fibrous cyst does not allow the normal drainage of the aqueous humor and creates pressure around the valve’s body.

A fibrous cyst appeared in seven patients that were subjected to needling; in four patients, the surgical excision of the cysts was made; in two patients at 1 month, in one at 6 month, and in the other at one year.

**Figure 2:**
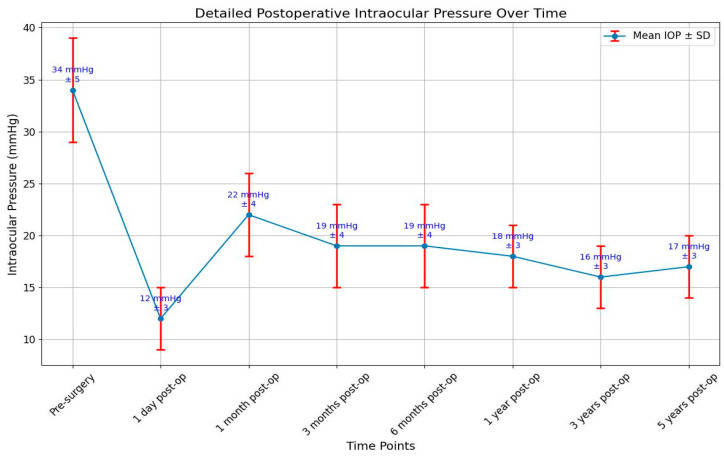
Intraocular pressure variations ± SD before and after surgery.

The wide range of IOP values (5–30 mmHg) underscores the need for individualized patient monitoring and management. The mean difference in IOP before and after surgery was found to be 7.53 mmHg. This indicates a statistically significant reduction in IOP post-surgery, supporting the effectiveness of the surgical technique employed. While some patients maintained IOP within the target range, others experienced either hypotonic or elevated pressures, highlighting the variability in postoperative outcomes. The skewed distribution towards higher IOP values suggests that a significant portion of measurements were at or above the target upper limit, which may indicate a need for more aggressive IOP management in some cases. The presence of both hypotonic and elevated pressures in the dataset emphasizes the importance of regular follow-up and potentially adjusting treatment strategies to achieve optimal IOP control (Figure 2).

According to the success and failure criteria defined before, finding an intraocular pressure above 22 mmHg in more than two visits that could not be controlled by medication, massage, needling, or excision of the cysts implied that the patient was in the failure category.

After one year postoperative, failure was declared in six patients (12%). They had elevated IOP during multiple visits due to the cyst formation and valve’s body entrapment. At the 3-year mark after surgery, another five patients developed high IOP due to this condition. And 5 years postoperative, valve body entrapment happened in another four patients, making it the most frequent complication (30%) leading to failure.

We concluded that the number of medications necessary for glaucoma control was reduced after surgery, from 3.37 before surgery to 1.21 after surgery. This decrease is noticed in most of the published data [9,10,11,12,13,14].

- Visual acuity variation after surgery

Visual acuity (VA) is the second criterion used to define successful or unsuccessful intervention. Loss of light perception or a decrease in VA of more than two rows in the optotype was considered a criteria for failure. Our study is a prospective one, allowing a good appreciation of the variation in visual acuity and a good appreciation of the evolution of lens-related problems. Visual acuity was measured using the Snellen optotype with the best correction. During the period of follow-up of a year, 8 patients (16%) out of 50 had a decrease in VA, and 3 of them had a decrease with more than two rows in the optotype, so the intervention was considered unsuccessful in those cases (Table 2).

The first patient, with glaucoma secondary to vitreoretinal surgery, had developed a valve capture with high-pressure values that could not be controlled; we practiced the excision of the fibrous cyst after a year, but the patient’s VA changed from LP (light perception) to WLP (without light perception).

The second patient, with neovascular secondary glaucoma, had a suprachoroidal hemorrhage and vitreous hemorrhage with a decrease in VA from LP to WLP.

The third patient with glaucoma secondary to vitreoretinal surgery, with surgically corrected retinal detachment, with surgically treated cataracts, silicone oil extraction, and iridectomy, developed suprachoroidal hemorrhage 3 months after surgery with a decrease in visual acuity from 0.05 to CF (counting fingers).

We noticed that all patients who lost light perception had a very low visual acuity at the beginning of the study, all of them being with only LP. We also noticed that in four patients (8%), visual acuity has increased. The patients had lens opacities before surgery that needed an extracapsular lens extraction with a pseudophakic implant after 6 to 12 months. In all three, the sight became better.

- Considerations regarding surgery complications

An old surgery axiom states that the best treatment for surgery complications is prevention. Each patient must be treated as a unique case.

Most of the complications appeared in the group of patients with glaucoma secondary to vitreoretinal surgery, them being best represented in this study. The second complication frequency was the subgroup of patients with secondary neovascular glaucoma (Figure 2).

Valve’s body entrapment was noticed in 15 (31.91%) patients, leading to an increase in intraocular pressure. Leaking of the aqueous humor in the space created between the tenon and sclera leads to an inflammatory reaction in the tenon with fibrous tissue development and modified collagen. A fibrous, hard capsule is formed around the valve’s body, and the filtering of the aqueous humor in the subconjunctival space is blocked; this leads to an increase in the pressure inside the valve. The high pressure around the body of the valve by the fibrous capsule leads to restriction on valve opening when intraocular pressure is above 8 mmHg, so the valve will not open when the pressure is even higher. It can be prevented by digital ocular massage, needling using an antimetabolite (5-fluorouracil), and regular check-ups [23,24].

Cyst excision was intended with local subconjunctival anesthesia. An incision at the level of the conjunctiva was made at the highest point of the cyst; the fibrous area behind the conjunctiva was extracted, and the valve stayed in place. After the cyst excision, the conjunctiva was sutured so that the valve was totally covered. The histologic exams of the peri-valve tissues extracted revealed modifications in collagen structure and transformations of fibroblasts to myofibroblast cells.

Hyphema appeared in seven (14.89%) patients a day after surgery. In six cases, hyphema resolved spontaneously in the next few days. In one of the patients, we practiced the washing of the anterior chamber and Avastin (Bevacizumab) injection in the anterior chamber.

Suture dehiscence was the third most frequent complication without exteriorization of the valve. This appeared in five (10.64%) patients. In three of these, after re-suturing, the evolution was good. In two patients at one year postoperative, a reintervention was needed, and the valve coverage with oral mucous tissue was performed. However, it proved to be inefficient as it reappeared, and we had to extract the valve between 1- and 3-years postoperative.

Tube blockage appeared in four patients (8.51%). In two patients, the tube was blocked by blood, and with the massage of the globe, the tube became patent, and there was no need for surgical intervention. In one patient, the iris blocked the tube, and a surgical intervention was needed to reposition the tube. In the fourth patient, the tube was blocked by a vitreous on the day following the surgery, with the patient having a vitreous in the anterior chamber. General and local treatment for glaucoma was intended but with no results. In 4 days after surgery, vitrectomy was intended because the high intraocular pressure could not be controlled.

Rebellious pain was unresponsive to any treatment developed in four (8.51%) patients. In the case of the patient with congenital glaucoma, the valve had to be explanted, while in other cases, the pain was remitted by itself.

Vitreous hemorrhage developed in three (6.38%) patients: two with neovascular glaucoma and one with aphakic glaucoma. In one patient, the hemorrhage resolved spontaneously, and in the other two, vitrectomy was needed.

Suprachoroidal hemorrhage developed in two (4.26%) patients: one with neovascular glaucoma and the other with glaucoma secondary to vitreoretinal surgery.

In the patient with neovascular glaucoma, visual acuity was reduced from LP to WLP. In the patient with secondary glaucoma after vitreoretinal surgery with surgically corrected retinal detachment and cataracts, silicone oil extraction, and iridectomy 3 months after the surgery, suprachoroidal hemorrhage developed, and visual acuity decreased from 0.05 to CF.

Retinal detachment developed in two (4.26%) patients with glaucoma secondary to vitreoretinal surgery in 4 and 6 months from the implant; surgical intervention was intended with re-attachment of the retina.

Diplopia developed in two (4.26%) patients. The first patient had secondary posttraumatic glaucoma. The valve was implanted in the super-nasal quadrant, and no intervention was needed because, at the next visit, the problem was solved. The second patient had glaucoma after vitreoretinal surgery, and the valve was implanted in the superotemporal quadrant. Diplopia developed when the patient was looking up and towards the right; in the primary position, there was no diplopia. The patient was satisfied, and the diplopia resolved spontaneously.

Hypotony occurred in one patient but resolved after injecting viscoelastic into the anterior chamber; there were no patients with athalamy. In the study group, I noticed a very small incidence rate of hypotonia. Unlike other artificial drainage systems, hypotonia is much less present in the AGV because of the valve mechanism. Unlike other studies, we obtained a much lower rate of hypotonia, and we believe that the implantation technique with the scleral flap is responsible for this; introducing the tube through the scleral flap makes a much better seal than covering the tube with preserved sclera.

Cornea-tube contact occurred in one patient; the tube was shortened on the second postoperative day without further damage to the cornea. At one year postoperative, one patient had endothelial damage and required a cornea transplant, after which the AGV tube was repositioned in the posterior chamber.

**Figure 3:**
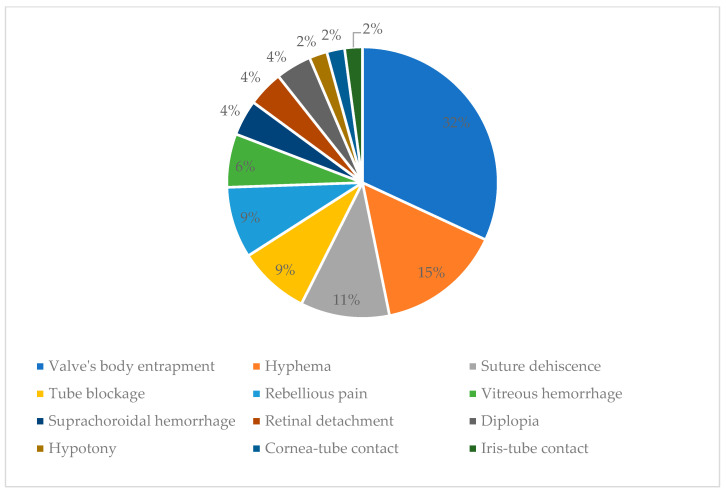
Surgery complications.

Iris-tube contact occurred in one patient a year after implant because the valve moved; we had to reposition the tube (Figure 3).

According to the success criteria we defined, we achieved a success rate of 82% one year after the implant. After 3 years, it was 68%, and after 5 years, it was 60%.

The predominance of the valve’s body entrapment as a cause of failure highlights a potential area for improvement in either the surgical technique or the design and handling of the Ahmed Valve. The variety of causes listed also underscores the complexity of the surgery and the need for comprehensive preoperative assessment and postoperative care to minimize risks. The data suggest that while the Ahmed Valve can be an effective treatment, there are specific complications that need to be addressed to improve overall success rates (Table 3).

Another objective of the study was to assess whether the rate of occurrence of postoperative complications after the valve implant in patients with previous antiglaucoma surgery is higher than the rate of complications in patients without previous glaucoma surgery. For this, we compared the complications and calculated if there is a correlation between the two variables.

The statistical correlation between the number of surgeries prior to the implant and postoperative complications was 0.876. The correlation is direct and strong, which means that it is statistically significant that patients with multiple ophthalmic surgeries had more frequent postoperative complications. The postoperative complications that occurred more frequently in patients with surgeries prior to the implant were wound dehiscence, valve entrapment, rebellious pain, hyphema, decreased visual acuity, and displaced tubes. The statistical correlation between the number of antiglaucoma surgeries prior to the implant and postoperative complications was 0.925. The correlation is direct and strong. The coefficient of correlation between patient age or area of origin and complications is negative; there is no statistically significant correlation between these two variables.

## 4. Discussion

The AGV represents a significant advancement in the surgical management of refractory glaucoma. This article examines the initial clinical experience with the AGV implant, focusing on several key areas: socio-demographic characteristics of the patient cohort, distribution of patients by glaucoma type, variations in IOP and visual acuity after surgery, and complications associated with the procedure.

Correlating the incidence of glaucoma with age and gender, we found that our patient cohort predominantly consisted of older adults, with a higher prevalence in males. This demographic trend aligns with the existing literature, which indicates that age and male gender are significant risk factors for glaucoma [2,6,22,25].

In this study, the majority of patients diagnosed with glaucoma had developed the condition secondary to vitreoretinal surgery, specifically due to the use of silicone oil for retinal detachment procedures. These patients experienced elevated IOP as a result of emulsified silicone oil blocking the pupil, causing iris synechia, or obstructing the camerular angle. This secondary glaucoma could not be managed with medication alone.

The second most common type of glaucoma in the study was primary open-angle glaucoma, affecting 20% of patients. These patients had not responded to medical treatments and other surgical interventions and showed progressive atrophy, necessitating the implantation of the AGV.

Neovascular glaucoma was the third most common, affecting 16% of patients. The relatively lower incidence of this type was attributed to the recent use of anti-VEGF agents, which have reduced the number of neovascular glaucoma cases requiring AGV implantation. All of these patients had failed trabeculectomy surgeries. After this study, we changed our therapeutic conduct, and now we implant AGV in secondary neovascular glaucoma per primam.

The targeted IOP post-surgery was set between 5 and 22 mmHg. Hypotonia, defined as an IOP below 5 mmHg, was observed in only one patient the day after AGV implantation, which was successfully corrected with viscoelastic injection. The use of a scleral flap to cover the tube likely contributed to the low incidence of hypotonia, a finding consistent with other studies.

The elastomeric membrane in the AGV helps maintain IOP above 8 mmHg, preventing hypotonia unless there are external losses. Elevated IOP (above 22 mmHg) was managed with medication, massage, needling, or surgical excision of fibrous cysts, which formed around the valve in some patients due to modified collagen from aqueous humor exposure.

Fibrous cysts requiring intervention were observed in seven patients, with varying follow-up times for surgical excision. Overall, the surgery reduced the number of medications needed for glaucoma control from an average of 3.37 to 1.21, aligning with published data.

In this study, the majority of complications occurred in patients with glaucoma secondary to vitreoretinal surgery, followed by those with neovascular glaucoma. The most frequent complications included hyphema, valve entrapment, suture dehiscence, tube blockage, rebellious pain, vitreous hemorrhage, suprachoroidal hemorrhage, retinal detachment, diplopia, hypotony, cornea–tube, and iris–tube contact. The study achieved a success rate of 82% one year after AGV implantation in the group, indicating a generally favorable outcome despite the noted complications. The developed complications were resolved either by re-intervening or spontaneously [26,27,28,29].

In comparison to the Tube Versus Trabeculectomy (TVT) study and the Primary Tube Versus Trabeculectomy (PTVT) study, our results show a similar effectiveness in IOP control and a comparable rate of complications. The TVT study, which focused on patients with prior ocular surgery, reported success rates and complication profiles similar to ours, with a notable difference being the type of implant used (Baerveldt versus Ahmed). However, the complication profile in our study showed some differences. Hyphema and valve body entrapment were notable complications in our cohort, particularly among patients with secondary glaucoma due to vitreoretinal surgery. These complications were less frequently highlighted in the TVT study, which may be due to differences in patient populations and surgical techniques. Our use of the AGV, which has a unique flow-restricting mechanism, might account for the lower incidence of hypotony compared to the Baerveldt implant used in the TVT study. The PTVT study, dealing with primary glaucoma cases, also showed effective IOP management but had fewer secondary glaucomas compared to our study. Our study’s patient population included a significant number of cases with secondary glaucoma, often post-vitreoretinal surgery, which contrasts with the primary glaucoma cases in the PTVT study. Despite these differences, our findings align with the PTVT study in demonstrating the efficacy of tube shunt surgery (Ahmed valve) in lowering IOP. In terms of postoperative interventions, our study reported several cases requiring additional surgeries, such as excision of fibrous capsules, cataract extractions, and vitrectomies. This is somewhat consistent with the PTVT study, where re-interventions were also necessary, though with different underlying causes and frequencies [9,10,11,12,13,14].

Recent advancements in glaucoma treatment, such as micro-pulse transscleral cyclophotocoagulation (MP-TSCPC) and continuous wave transscleral cyclophotocoagulation (CW-TSCPC), have shown promising results in managing IOP in neovascular glaucoma. Zemba et al. (2022) compared these two methods, finding that MP-TSCPC offered better IOP control and fewer complications than CW-TSCPC in NVG patients. These findings are relevant to our study as they highlight alternative or adjunctive treatments that could potentially enhance outcomes for patients with refractory glaucoma, particularly those with neovascular glaucoma. Incorporating such advanced treatment options could further improve the efficacy and safety profile of glaucoma management strategies, offering additional hope for patients who do not respond to conventional therapies [30,31,32,33].

Another issue that we encountered was patient compliance. Indeed, during the follow-up period, we experienced some cases of missing visits and missing data. Specifically, three patients dropped out of the study due to death, and five patients missed scheduled follow-up visits due to various reasons. Throughout the study, we maintained close contact with our patients, utilizing phone calls, communication with family members, and offering free visits to encourage participation and adherence to the follow-up schedule. Despite these efforts, some data gaps remained due to the reasons mentioned above. However, we believe that the overall impact on our study’s findings was minimal and that the strategies we employed helped to maintain the integrity of our data as much as possible. In order to minimize the occurrence of missing visits and data in future studies, several strategies can be implemented, such as electronic reminders, home visits for patients who are unable to attend visits due to mobility issues, offering flexible scheduling options for follow-ups, and enhancing patient engagement through education about the study’s importance and their role in it can foster a sense of commitment and responsibility [32,33,34].

In addition, it is important to note that we did not track the visual field as a parameter in this study. The primary focus was on reducing intraocular pressure, as the included patients already had significantly low visual acuity and visual field at the beginning of the study.

Furthermore, we have not investigated the number of corneal endothelial cells, which is a limitation. We currently plan to address this and investigate the gonioscopic position of the tube in the anterior chamber, assessing whether it is closer or farther from the corneal endothelium.

Analyzing the trends and patterns observed in this study suggests several underlying mechanisms and relationships. The high incidence of secondary glaucoma post-vitreoretinal surgery indicates a strong association between retinal interventions and subsequent glaucoma development. The significant reduction in IOP and medication dependency post-AGV implantation underscores the valve’s effectiveness in managing refractory cases. Moreover, the complications profile highlights the need for meticulous surgical techniques and postoperative management to mitigate risks. The correlation between patient demographics and glaucoma incidence emphasizes the importance of tailored treatment approaches based on age and gender, acknowledging these as critical factors in disease progression and management outcomes.

The prognosis of patients after the 5-year postoperative period was generally positive, with sustained IOP control and reduced medication dependency. However, long-term monitoring is crucial to manage potential late-onset complications and to ensure the continued success of the AGV implantation.

The study highlights the complication rate in these groups and outlines various corrective procedures performed to manage these issues. The findings contribute to understanding the effectiveness and challenges of AGV implantation, suggesting future research directions to improve patient outcomes and reduce complication rates.

## 5. Conclusions

1. The mean number of glaucoma medications used postoperatively is lower; the average in the adult group decreased from 3.37 preoperatively to 1.21 postoperatively.
2. According to the success and failure criteria, a year after the AGV implant, success was recorded in 41 adult patients, with the success rate being 82% at one year, 68% after 3 years, and 60% after 5 years postoperative.
3. AGV is a solution not only for refractory glaucoma after classic surgery but can be used for secondary glaucoma after vitreoretinal surgery or for neovascular glaucoma refractory to drug therapy. It might be the first indication of antiglaucoma surgery in these patients, with a high success rate.
4. Subconjunctival anesthesia is a good option for glaucoma surgery, including AGV insertion, providing good mobility of the eye and no need for a traction suture.
5. AGV can be used as the first option of surgical treatment in secondary neovascular glaucoma, with classic trabeculectomy having a high risk of failure.
6. Even though it is a difficult procedure, with good surgical technique and careful and frequent patient follow-up, in the long run, AGV is a chance for patients with difficult glaucoma to keep their vision.

## Figures and Tables

**Table 1:** Distribution of patients by glaucoma type.

Glaucoma Type	Patients n.	%
Total	50	100%
Secondary glaucoma after vitreoretinal surgery	12	24%
Primary open-angle glaucoma	10	20%
Neovascular secondary glaucoma	8	16%
Primary closed-angle glaucoma	7	14%
Aphakic secondary glaucoma	6	12%
Traumatic secondary glaucoma	2	4%
Congenital glaucoma	1	2%
Uveitic secondary glaucoma	1	2%
Juvenile glaucoma	1	2%
Secondary glaucoma after keratoplasty	1	2%
Posterior embryotoxon	1	2%

**Table 2:** Decrease in sight by glaucoma type at 1 year postoperative.

Type of Glaucoma with a Decrease in Sight	Number of Patients with a Decrease in VA	Number of Patients with a Decrease in VA of More Than 2 Rows	Number of Patients with VA = WLP
Glaucoma secondary to vitreoretinal surgery	3	1	1
Secondary neovascular glaucoma	2	0	1
Primary glaucoma with open angle	2	0	0
Juvenile glaucoma	1	0	0
Total	8	1	2

**Table 3:** Patients who experienced failure at 5 years postoperative.

Cause	Nr. of Failed Cases
Valve’s body entrapment	15
Suprachoroidal haemorrhage	2
Suture dehiscence	2
Rebellious pain	1

## Data Availability

The original contributions presented in the study are included in the article; further inquiries can be directed to the corresponding author/s.
